# Psychometric Evaluation of the Beliefs About Stress Scale in the German General Population

**DOI:** 10.1002/mpr.70026

**Published:** 2025-06-14

**Authors:** Bjarne Schmalbach, Ileana Schmalbach, Johannes A. C. Laferton, Bernhard Strauß, Jörg M. Fegert, Elmar Brähler, Katja Petrowski

**Affiliations:** ^1^ Medical Psychology and Medical Sociology University Medical Center of the Johannes Gutenberg‐University Mainz Mainz Germany; ^2^ Institute for Mental Health and Behavioral Medicine & Department of Medicine HMU Health and Medical University Potsdam Potsdam Germany; ^3^ Institute of Psychosocial Medicine Psychotherapy and Psychooncology Jena University Hospital Friedrich Schiller‐University Jena Jena Germany; ^4^ Department for Child and Adolescent Psychiatry Psychotherapy University of Ulm Ulm Germany; ^5^ Department of Medical Psychology and Medical Sociology University Medical Center Leipzig Leipzig Germany

## Abstract

**Background:**

Beliefs about stress significantly influence health outcomes. Reliable, economical scales are essential for assessing these beliefs to foster understanding on mechanisms by which stress mindsets affect health outcomes. Such understanding can inform interventions promoting adaptive stress perceptions and reducing chronic stress‐related health risks.

**Method:**

This study assessed the Beliefs About Stress Scale (BASS) in a representative German sample (*N* = 2515). We conducted confirmatory factor analysis to evaluate factorial validity and tested measurement invariance was tested across demographics, and the BASS's associations with related constructs were analyzed for convergent and discriminant validity.

**Results:**

The BASS demonstrated strong factorial validity, with a bifactor model offering superior fit to the three‐factor structure. Measurement invariance analyses confirmed stability across demographics, though minor age‐specific adjustments may improve interpretability. Positive beliefs about stress controllability and benefits were associated with healthier psychological outcomes, whereas negative stress beliefs linked to poorer mental health, underscoring the value of a “stress‐is‐enhancing” mindset. Normative data enhance the BASS's applicability in both research and clinical settings.

**Conclusion:**

The BASS exhibits robust validity and reliability, making it suitable for population‐level applications and comparisons between groups. By clarifying the influence of stress mindsets on stress regulation, the BASS supports the development of mindset‐based interventions that promote adaptive stress perceptions and help mitigate chronic stress risks.

## 1 Introduction

Past evidence highlights major increases in stress‐related health concerns from 2019 to 2023 (APA 2023). For instance, epidemiological data shows a 42% rise in stress‐related mental disorders (APA 2023; Chourpiliadis et al. 2024; Reed et al. 2021; Tian et al. 2022). These trends underscore the need for systematic stress assessment to clarify how stress contributes to escalating health burden.

Stress arises when individuals appraise environmental demands as surpassing their resources, triggering a cascade of emotional (e.g., fear) and behavioral (e.g., avoidance) responses that are often accompanied by physiological changes, such as heightened hypothalamic‐pituitary‐adrenal (HPA) axis engagement (Lazarus 1984; Juruena et al. 2020; Seery 2011). While acute stress responses can be adaptive, enabling the body to respond effectively to immediate threats, prolonged stress exposure is associated with numerous mental and somatic health concerns (Agorastos and Chrousos 2022; Schneiderman et al. 2005; McEwen 1998; Fischer and Nater 2020). This established pathogenic trajectory necessitates elucidating the cognitive mechanisms that modulate stress responses and their consequent health outcomes.

Cognitive appraisals, which refer to the mental evaluation of stressors, serve as pivotal mediators between objective stressors and the individual's stress response, influencing emotional, physiological, and behavioral reactions to stress (Junker et al. 2021; Phelps 2006; Jamieson et al. 2018; Gaab 2009). Research indicates that stress beliefs or “stress mindset”—the belief that stress is either enhancing or debilitating—profoundly shape stress responses and subsequent health outcomes (Crum et al. 2013; Crum et al. 2023). Past evidence suggests that an adaptive “stress‐is‐enhancing” mindset has been linked to better performance, improved health, and psychological resilience, as well as an internal locus of control and moderated cortisol responses (Keech et al. 2018; J. A. Laferton et al. 2023; J. A. C. Laferton et al. 2024; Uzun and Karatas 2020; Yeager et al. 2022). Conversely, negative beliefs about stress are associated with deleterious health outcomes, including increased risks of cardiovascular morbidity and mortality (Nabi et al. 2013). The clinical importance of these findings is further underscored by Crum et al. (2023) showing that individuals with a “stress‐is‐debilitating” mindset exhibited worse cognitive and affective outcomes. Similarly, Keller et al. (2012) documented elevated mortality risk among individuals who both experienced high stress levels and perceived stress as harmful to their health.

The absence of psychometrically robust, population‐validated stress belief measures represents a critical barrier to advancing stress research and developing effective interventions. While the Stress Mindset Measure (SMM; Crum et al. 2013) and the Beliefs About Stress Scale (BASS; J. A. Laferton et al. 2018) demonstrate promising preliminary psychometric properties, both instruments exhibit methodological limitations that constrain their broader implementation in clinical and research settings. Existing validation studies of these most commonly used tools (SMM & BASS; see Kilby et al. 2020 for further relevant questionnaires) have exclusively relied on convenience samples (e.g. students) substantially limiting their generalizability across diverse populations and reducing their clinical utility. To date, neither has been examined in a representative sample, nor do they offer normative data necessary for individual diagnostic applications and consequently clinical interpretation remains limited.

The BASS holds particular promise compared to other instruments due to its comprehensive approach (J. A. Laferton et al. 2018), identifying three dimensions: negative stress beliefs, positive stress beliefs, and controllability. Its initial validation was conducted in a sample of German university students (J. A. Laferton et al. 2018) and further factorial and predictive validity was established in specific samples (e.g. nurses, physicians, students; Bai and Bai 2024; J. A. C. Laferton et al. 2024; Ma et al. 2023; Wekenborg et al. 2024; Wen‐feng 2019). Hence, it lacks validation in representative population samples, preventing the establishment of normative benchmarks essential for several critical applications: (1) accurate individual assessment and clinical interpretation in diverse populations, (2) identifying at‐risk subgroups with maladaptive stress beliefs for targeted interventions, (3) evaluating intervention efficacy with standardized, generalizable metrics, and (4) facilitating cross‐cultural and cross‐population comparisons of stress belief distributions. This methodological gap has hampered progress in understanding population‐level patterns of stress beliefs and their relationship to health disparities and outcomes. Therefore, the present study addresses this critical gap by conducting the first comprehensive psychometric evaluation of the BASS in demographically representative German population sample (*N* = 2515) thereby establishing normative benchmarks and advancing both theoretical understanding and clinical applications of stress belief assessment. Specifically, we examined (1) correlations with convergent measures, (2) factorial validity, and (3) group differences by sex and age. Additionally, normative data in percentile ranks are provided. First, to assess convergent and discriminant validity, we calculated correlations between the BASS and established measures of mental health, control beliefs, and stress. We hypothesize that negative stress beliefs will positively correlate with depression, anxiety, external locus of control, work‐related problems, and perceived stress, while negatively correlating with internal locus of control and sense of coherence. We expect that beliefs in stress controllability will be positively associated with internal locus of control and sense of coherence and negatively associated with depression, anxiety, external locus of control, work‐related problems, and perceived stress. Lastly, we hypothesize that positive beliefs about stress will positively correlate with internal locus of control and sense of coherence and show negative associations with depression, anxiety, external locus of control, work‐related problems, and perceived stress.

## 2 Method

### 2.1 Sample and Procedure

The study utilized a representative sample of the German adult population collected in 2023. Participants were recruited with the assistance of a German market research agency (Unabhängiger Service für Umfragen, Methoden und Analysen, Berlin, Germany) through random sampling to ensure diversity across sociodemographic variables. Data collection adhered to ethical guidelines based on the Declaration of Helsinki and approval from the ethics committee of the University of Leipzig (063–02010.14–10032014), with informed consent obtained from all participants prior to participation. All participants were visited at home by a trained interviewer.

### 2.2 Sample Characteristics

The sample was balanced in terms of sex, with 53% female (*n* = 1325). The mean age of participants was 50 years (SD = 18). Marital status was varied, with most of the population being married (44% married; *n* = 1094). Educational background indicated that 26% had less than 10 years of schooling, 46% completed 10 years, 26% had more than 10 years, and 1% were currently students. For further characteristics see Table 1.

**TABLE 1:** Sample description (*N* = 2515).

Variable	*n* (%)
Sex	
Female	1325 (53)
Male	1189 (47)
Diverse	1 (0)
Age, M(SD)	50 (18)
< 30 years	424 (17)
30–39.99 years	359 (14)
40–49.99 years	386 (15)
50–59.99 years	530 (21)
60–69.99 years	435 (17)
≥ 70 years	381 (15)
Marital status	
Married	1094 (44)
Separated	43 (2)
Single	757 (30)
Divorced	361 (14)
Widowed	256 (10)
Educational background	
< 10 years	666 (26)
10 years	1156 (46)
> 10 years	653 (26)
Currently student	36 (1)
Net household income per month	
< 2000€	754 (30)
20.0–3499€	1022 (41)
35.0–4999€	493 (20)
≥ 5000€	208 (8)

### 2.3 Instruments

Beliefs about stress scale (BASS; J. A. Laferton et al. 2018). This self‐report questionnaire was implemented to evaluate different beliefs about stress. The BASS assesses three dimensions based on 15 items related to stress beliefs: negative stress beliefs (BASS‐N), positive stress beliefs (BASS‐P), and perceived control over stress (BASS‐C). Sample items include “Being stressed drains a good deal of my energy” (BASS‐N), “Being stressed activates my ressources” (BASS‐P), and “Being stressed is something I am able to control to a certain degree” (BASS‐C). Responses are scored on a four‐point Likert scale, from 1 (completely disagree) to 4 (definitely agree).

Internal‐External Locus of Control shortscale (IE‐4; Kovaleva et al. 2012). The IE‐4 was used to measure the locus of control, that is, beliefs about whether the outcomes of one's actions are shaped by own efforts or by external forces beyond one's influence. The scale is based on four items capturing two dimensions: internal LoC (LoC‐I) and external LoC (LoC‐E) describing beliefs of personal control. Sample items include “I am my own boss” (LoC‐I), “Fate often gets in the way of my plans” (LoC‐E). The items range from 1 (does not apply at all) to 5 (applies completely). Reliability as assessed by *ω* was acceptable for the Internal, *ω* = 0.768, and the External subscale, *ω* = 0.706.

The Sence of Coherence shortscale (SOC‐9; Schmalbach et al. 2020; Schumann et al. 2003) was used to determine on the person's capacity to respond to illness and treatment based on Antonovsky's sense of coherence (1985). The scale consists of nine items (e.g. “How often do your feelings and thoughts feel completely mixed up?”) ranging from 1 (never) to 3 (very often). Reliability in the present sample was very good, *ω* = 0.924.

Patient Health Questionare‐4 (PHQ‐4; Kroenke et al. 2009; Löwe et al. 2010; Wicke et al. 2022) was used as a screening tool for depression and anxiety. The PHQ‐4 is a self‐report scale comprising four items that align with the criteria for major depressive disorder and generalized anxiety disorder as outlined in the Diagnostic and Statistical Manual of Mental Disorders (DSM‐IV). Each item requires respondents to rate the extent to which they have experienced specific depressive symptoms over the past 2 weeks. Sample item include “Little interest or pleasure in doing things”. Ratings range from 0 (Not at all) to 3 (Nearly every day). Reliability in the present sample was good for the depression, *ω* = 0.838., as well as the anxiety subscale, *ω* = 0.825.

The Perceived Stress Scale (PSS‐4; Schmalbach et al. 2025), originally developed by Cohen et al. (1983), is a widely recognized self‐report tool grounded in the psychological framework of stress. The four‐item measure assesses the extent to which individuals have perceived their lives over the past month as unpredictable, uncontrollable, and overwhelming (e.g. “have you felt that things were going your way?”). Responses are recorded on a five‐point scale ranging from 0 (never) to 4 (very often), with intermediate options of 1 (almost never), 2 (sometimes), and 3 (fairly often). McDonald's *ω* was 0.815 in the present samle.

### 2.4 Statistical Analyses

All analyses for the present study were carried in R, using the packages lavaan and semTools (Jorgensen et al. 2022; Rosseel 2012). Since the item‐level distributions cannot be considered interval‐scaled or normal, we conducted confirmatory factor analysis for ordinal indicators using the robust weighted least squares estimator (WLSMV; Li 2016). Accordingly, only complete data was utilized in the analyses. Missingness was low across all values of the BASS (0.4%), but since even a single missing value leads to exclusion 103 individuals had to be excluded, leading to a sample size of *n* = 2412.

We evaluated to models: the common three‐factor model and the bifactor model with three specific subfactors (both without any error term correlations). To this end, we utilized the χ^2^‐test, along with the common fit indices: Comparative Fit Index (CFI), Tucker‐Lewis Index (TLI), Root Mean Square Error of Approximation (RMSEA), and Standardized Root Mean Square Residual (SRMR). According to the traditional rules by Hu and Bentler (1999), CFI and TLI should be greater than 0.95, whereas RMSEA and SRMR should be smaller than 0.06 and 0.08, respectively. In addition, we estimate internal consistency based on McDonald's *ω* (Dunn et al. 2014; Rodriguez et al. 2016).

In order to evaluate measurement invariance, we followed the steps laid out by Wu and Estabrook (2016): First we constrained item thresholds to be equal across groups, second, we constrained factor loadings, and third we constrained item intercepts. Between each of these successive steps we conducted a χ^2^‐test and in addition evaluated the change in CFI and RMSEA. The latter two should not worsen by more than 0.010 and 0.015, respectively (Chen 2007; Cheung and Rensvold 2002).

## 3 Results

### 3.1 Factorial Validity

The three‐factorial model of the BASS had mixed fit results, χ^2^ (87) = 2670.09, *p* < 0.001, CFI = 0.956, TLI = 0.947, RMSEA = 0.111, SRMR = 0.062. Specifically, CFI and SRMR were acceptable, whereas TLI and RMSEA were not. Factor loadings are displayed in Table 2. Reliability was good to very good for all three scales: *ω*_Negative Stress Beliefs_ = 0.924, *ω*_Controllability Beliefs_ = 0.801 *ω*_Positive Stress Beliefs_ = 0.909. For comparative purposes Cronbach's *α* was 0.890, 0.804, and 0.905 for the three scales. Average Variance Extracted amounted to 0.608, 0.666, and 0.791, respectively. Heterotrait‐Monotrait ratios (HTMT) were between 0.322 and 0.604 for the three latent variables.

**TABLE 2:** Item descriptive statistics.

Item	Relative response frequency in %					*λ*_Bifactor_	
	1	2	3	4	*λ*_3FM_	General	Specific
1^N^	3	10	44	43	0.713	0.414	0.598
2^N,r^	15	34	38	12	0.641	0.600	0.212
3^N^	7	26	39	28	0.778	0.512	0.576
4^N^	4	15	43	38	0.774	0.460	0.635
5^N^	7	30	40	23	0.847	0.515	0.680
6^N^	8	27	41	24	0.903	0.563	0.702
7^N^	6	18	38	38	0.811	0.428	0.721
8^N^	11	30	36	22	0.740	0.383	0.671
9^C^	9	29	49	13	0.705	−0.353	0.687
10^C^	10	30	46	14	0.894	−0.514	0.747
11^C^	6	25	57	12	0.838	−0.544	0.555
12^P^	21	44	28	7	0.911	−0.933	−0.046
13^P^	23	43	28	7	0.912	−0.905	0.061
14^P^	27	41	25	7	0.904	−0.870	0.400
15^P^	19	33	37	10	0.827	−0.780	0.305

Abbreviations: C = Controllability Beliefs; N = Negative Stress Beliefs; P = Positive Stress Beliefs; r = Item is reverse‐coded.

In an effort to find a better fitting solution, we also tested a bifactorial model, which ‐ in addition to the above‐mentioned three specific factors—adds a general factor. The correlations between all latent variables in this model are set to 0. The resultant model fit was significantly improved over the standard correlated‐factors solution, χ^2^ (75) = 1047.47, *p* < 0.001, CFI = 0.984, TLI = 0.977, RMSEA = 0.073, SRMR = 0.033. All fit indices were acceptable, even good. We then calculated *ω* according to Rodriguez et al. (2016), which helps with attributing the portions of variance to general and specific factors. Specifically, we first calculated *ω*_Total_ at 0.957, which represents the total proportion of systematic variance in the data. Second, we determined *ω*_Hierarchical_ at 0.705, which represents the proportion of variance explained by the general factor in the data. This indicates that the vast majority of variance is attributable to a general construct—as opposed to a specific one. Finally, we calculated *ω*_Subscale_ for each of the specific factors. This version of the coefficient partials out any variation already explained by the general factor. Here we found that the majority of specific factor variation is contained in the Negative Stress Beliefs specific factor: *ω*_Negative Stress Beliefs_ = 0.712, *ω*_Controllability Beliefs_ = 0.122 *ω*_Positive Stress Beliefs_ = 0.020. These coefficients indicate that out of the three specific facors, only the one dealing with Negative Stress Beliefs covers any meaningful variation on top of the general factor. Average Variance Extracted mirrors these results: AVE_Total_ = 0.688, AVE_G_ = 0.377, AVE_Negative Stress Beliefs_ = 0.204, AVE_Controllability Beliefs_ = 0.089, AVE_Positive Stress Beliefs_ = 0.017.

### 3.2 Convergent Validity

As reported in Tables 3 and 4, we calculated the latent correlations for the correlated factors model and the bifactorial model of the BASS with conceptually related scales. In summary, the analyzed effects were of moderate magnitude, indicating substantial overlap between the constructs under study. Specifically, the presented results in Table 3, 4 show that BASS‐N is positively correlated with PHQ‐Depression and PHQ‐Anxiety, IE‐4 E, Work Problems and PSS, while negatively associated with IE‐4 I and SOC. The dimension BASS‐C is positively associated with IE‐4 and SOC, and negatively related to PHQ‐Depression and PHQ‐Anxiety, IE‐4 E, work‐related problems, and PSS. Similarly, BASS‐P demonstrated positive correlations with IE‐4 I and SOC, and negative associations with PHQ‐Depression and PHQ‐Anxiety, IE‐4 E, and PSS. The general factor in the bifactor model had a similar pattern of correlations as the BASS‐N subscale in the correlated factors model, only higher in magnitude. The specific factors were for the most part negligible, except for the BASS‐P factor which was also similar to the general factor. This result is surprising since it is a reversal from the original pattern in the correlated factors model but it emphasizes the difference between a congeneric factor and the specific subscale factor—which is simply a remainder of variance after the modeling of the general factor.

**TABLE 3:** Latent correlation matrix, correlated factors model.

	BASS‐N	BASS‐C	BASS‐P	PHQ dep	PHQ anx	IE‐4 int	IE‐4 ext	SOC	Work problems	PSS
BASS‐N	1									
BASS‐C	−0.376	1								
BASS‐P	−0.614	0.565	1							
PHQ dep	0.342	−0.334	−0.274	1						
PHQ anx	0.300	−0.303	−0.238	0.922	1					
IE‐4 internal	−0.247	0.416	0.310	−0.571	−0.517	1				
IE‐4 external	0.226	−0.319	−0.220	0.493	0.481	−0.547	1			
SOC	−0.258	0.420	0.280	−0.807	−0.755	0.690	−0.597	1		
PSS	0.174	−0.273	−0.176	0.481	0.458	−0.430	0.417	−0.544	0.318	1

*Note:* All correlations were highly significant at *p* < 0.001.

Abbreviations: BASS = Beliefs About Stress Scale; C = Controllability Beliefs; IE‐4 Ext = Internal–External Locus of Control Short Scale–4 External subscale; IE‐4 Int = Internal–External Locus of Control Short Scale–4 Internal subscale; N = Negative Stress Beliefs; P = Positive Stress Beliefs; PHQ Anx = Patient Health Questionnaire Anxiety Subscale; PHQ Dep = Patient Health Questionnaire Depression subscale; PSS = Perceived Stress Scale; SOC = Sense of Coherence.

**TABLE 4:** Latent correlation matrix, bifactorial model.

	BASS‐G	BASS‐N	BASS‐C	BASS‐P	PHQ dep	PHQ anx	IE‐4 int	IE‐4 ext	SOC	PSS
BASS‐G	1									
BASS‐N	0	1								
BASS‐C	0	0	1							
BASS‐P	0	0	0	1						
PHQ dep	0.479	0.042	−0.064	0.557	1					
PHQ anx	0.458	0.004	−0.039	0.609	0.922	1				
IE‐4 internal	−0.510	0.107	0.142	−0.541	−0.571	−0.517	1			
IE‐4 external	0.298	0.044	−0.179	0.190	0.493	0.481	−0.548	1		
SOC	−0.533	0.113	0.133	−0.702	−0.806	−0.755	0.690	−0.597	1	
PSS	0.347	−0.065	−0.086	0.473	0.481	0.458	−0.430	0.417	−0.544	1

*Note:* All correlations were highly significant at *p* < 0.001.

Abbreviations: BASS = Beliefs About Stress Scale; C = Controllability Beliefs; G = General Factor; IE‐4 Ext = Internal–External Locus of Control Short Scale–4 External subscale; IE‐4 Int = Internal–External Locus of Control Short Scale–4 Internal subscale; N = Negative Stress Beliefs; P = Positive Stress Beliefs; PHQ Anx = Patient Health Questionnaire Anxiety Subscale; PHQ Dep = Patient Health Questionnaire Depression subscale; PSS = Perceived Stress Scale; SOC = Sense of Coherence.

### 3.3 Group Comparisons and Norm Values

Initially, we conducted multigroup confirmatory factor analyses to ensure that the measurement models for the BASS are equivalent for the compared grouping variables. As reported in Tables 5 and 6, this is largely the case. Specifically, threshold invariance held for both models and both grouping variables. Loading invariance, on the other hand, can be assumed when comparing the sexes, but seems at least questionable when comparing age groups. For the bifactor model, ΔCFI indicated a significant deviation in addition to the significant χ^2^‐test, whereas it was barely acceptable for the correlated factors model. In contrast, there is good evidence for the invariance of indicator intercepts across both grouping variables and models.

**TABLE 5:** Multigroup factor analysis, correlated factors model.

Model	χ^2^	df	Δχ^2^	Δ*df*	*p*	CFI	ΔCFI	RMSEA	ΔRMSEA	Effect size
Sex										*d* [N, C, P]
Free model	2788.14	141				0.954		0.087		
Equal: Τ	2848.45	186	6.31	45	0.063	0.954	0.000	0.076	0.011	
Equal: Τ, Λ	2985.43	201	136.98	15	< 0.001	0.952	0.002	0.074	0.001	
Equal: Τ, Λ, *ν*	3023.73	213	38.31	12	< 0.001	0.952	0.000	0.073	0.002	
Equal: Τ, Λ, *ν*, *α*	3702.53	216	678.79	3	< 0.001	0.940	0.012	0.080	0.008	0.301, −0.012, −0.194
Age										*R* ^*2*^ [N, C, P]
Free model	3283.86	417				0.951		0.052		
Equal: Τ	3488.34	642	204.48	225	0.833	0.951	0.000	0.042	0.010	
Equal: Τ, Λ	4002.43	717	514.10	75	< 0.001	0.944	0.008	0.043	0.001	
Equal: Τ, Λ, *ν*	4130.97	777	128.54	60	< 0.001	0.942	0.001	0.042	0.001	
Equal: Τ, Λ, *ν*, *α*	6356.20	792	2225.23	15	< 0.001	0.904	0.038	0.053	0.011	0.062, 0.027, 0.035

Abbreviations: *α* = Latent means; Λ = Factor loadings; C = Controllability Beliefs; CFI = Comparative Fit Index; N = Negative Stress Beliefs; P = Positive Stress Beliefs; RMSEA = Root Mean Squared Error of Approximation; *ν* = Item intercepts; Τ = Item thresholds.

**TABLE 6:** Multigroup factor analysis, bifactorial model.

Model	χ^2^	df	Δχ^2^	Δ*df*	*p*	CFI	ΔCFI	RMSEA	ΔRMSEA	Effect size
Sex										*d* [N, C, P, G]
Free model	919.66	115				0.986		0.053		
Equal: Τ	979.97	160	6.31	45	0.063	0.986	0.000	0.045	0.008	
Equal: Τ, Λ	1125.14	190	145.17	30	< 0.001	0.984	0.002	0.044	0.001	
Equal: Τ, Λ, *ν*	1168.23	201	43.087	11	< 0.001	0.983	0.001	0.044	0.000	
Equal: Τ, Λ, *ν*, *α*	1842.24	205	674.012	4	< 0.001	0.972	0.012	0.057	0.013	0.203, 0.140, 0.003, 0.216
Age										*R* ^*2*^ [N, C, P, G]
Free model	1167.82	339				0.986	NA	0.031	NA	
Equal: Τ	1372.30	564	204.48	225	0.833	0.986	0.000	0.024	0.007	
Equal: Τ, Λ	2373.74	714	1001.44	150	< 0.001	0.971	0.015	0.030	0.006	
Equal: Τ, Λ, *ν*	2485.49	769	111.75	55	< 0.001	0.970	0.001	0.030	0.000	
Equal: Τ, Λ, *ν*, *α*	4727.51	789	2242.02	20	< 0.001	0.932	0.038	0.045	0.015	0.046, 0.013, 0.033, 0.029

Abbreviations: α = Latent means; Λ = Factor loadings; C = Controllability Beliefs; CFI = Comparative Fit Index; G = General Factor; N = Negative Stress Beliefs; P = Positive Stress Beliefs; RMSEA = Root Mean Squared Error of Approximation; ν = Item intercepts; Τ = Item thresholds.

In the next step, we constrained the latent variable means to be equal between groups. Here we found larger deviations compared to those resulting from constraints of the measurement model reported above. In order to allow for a standardized evaluation of the associated effect sizes we calculated the standardized mean difference score in the sense of Cohen's *d* and variance explained in the sense of *R* ^*2*^, respectively. In summary, the analyzed effects were of small to moderate magnitude both when comparing sexes and when comparing across age groups. In Tables 7 and 8 we provide normative percentile ranks of the German general population.

**TABLE 7:** Percentile ranks for three‐factor model, male.

Age group, years	18–29			30–39			40–49			50–59			60–69			≥ 70		
Sum score	N	C	P	N	C	P	N	C	P	N	C	P	N	C	P	N	C	P
3		3			2			5			3			3			4	
4		5	8		2	7		12	16		8	10		5	9		5	9
5		7	11		9	12		16	20		12	16		7	10		10	15
6		19	16		22	15		27	28		26	23		14	13		20	19
7		34	22		34	24		42	34		37	27		30	17		33	25
8	3	48	39	1	43	41	1	64	56	1	54	54	2	46	31	0	46	47
9	4	84	46	2	77	55	1	88	64	2	84	64	2	76	43	2	77	58
10	7	89	56	2	90	65	2	96	74	2	91	74	6	91	54	2	88	68
11	9	96	62	3	95	76	4	98	82	3	96	80	8	95	65	4	94	73
12	12	100	83	4	100	91	5	100	95	3	100	90	9	100	85	6	100	90
13	14		87	5		94	6		98	4		92	13		90	8		94
14	19		89	9		96	10		99	5		94	16		93	11		96
15	23		92	13		97	14		99	9		95	21		96	14		97
16	29		100	20		100	19		100	13		100	27		100	21		100
17	36			26			23			17			31			24		
18	43			35			29			21			39			32		
19	49			40			35			25			50			40		
20	55			53			45			30			58			48		
21	61			60			57			35			65			56		
22	71			65			65			43			73			64		
23	76			72			69			54			78			70		
24	82			80			76			63			85			77		
25	85			83			80			67			87			82		
26	87			86			85			75			90			86		
27	90			91			91			80			91			90		
28	93			94			94			89			92			94		
29	96			97			100			94			95			96		
30	97			100						97			98			100		
31	100									100			100					
32																		

Abbreviations: C = Controllability Beliefs; N = Negative Stress Beliefs; P = Positive Stress Beliefs.

**TABLE 8:** Percentile ranks for three‐factor model, female.

Age group, years	18–29			30–39			40–49			50–59			60–69			≥ 70		
Sum score	N	C	P	N	C	P	N	C	P	N	C	P	N	C	P	N	C	P
3		4			1			9			4			1			3	
4		6	15		2	9		12	22		7	18		4	7		5	12
5		9	21		7	13		16	30		11	26		9	11		9	18
6		19	25		20	19		39	36		23	32		18	19		20	24
7		32	29		32	24		52	43		36	40		32	23		32	32
8	1	51	45	1	42	44	1	66	66	0	58	62	1	49	45	0	49	52
9	2	80	57	2	82	55	1	89	75	2	83	73	3	78	55	1	81	63
10	4	85	68	3	89	65	2	94	81	4	90	79	4	88	68	1	89	72
11	5	92	75	4	96	73	3	99	86	6	94	84	6	93	77	2	94	83
12	6	100	90	5	100	88	4	100	95	8	100	96	6	100	87	3	100	91
13	8		93	9		90	5		97	12		97	8		88	3		94
14	12		94	11		92	7		99	17		98	10		92	6		96
15	19		96	15		94	11		99	19		98	11		95	9		96
16	23		100	23		100	13		100	21		100	12		100	14		100
17	26			26			15			24			16			18		
18	33			30			18			30			24			23		
19	37			35			22			42			27			30		
20	43			41			28			48			34			37		
21	52			49			40			57			38			44		
22	58			59			45			63			45			54		
23	66			67			51			69			58			65		
24	72			74			56			78			69			69		
25	78			80			62			87			74			76		
26	83			83			71			91			80			83		
27	90			89			78			100			86			89		
28	95			92			83						89			96		
29	97			96			91						93			100		
30	100			100			100						96					
31													99					
32																		

Abbreviations: C = Controllability Beliefs; N = Negative Stress Beliefs; P = Positive Stress Beliefs.

## 4 Discussion

The present study represents the first comprehensive evaluation of the psychometric properties of the BASS within a large representative German population sample. This validation addresses a methodological gap in stress assessment scales by specifically examining (1) correlations with convergent measures, (2) factorial validity, and (3) measurement invariance across sex and age, addressing methodological gaps in the stress assessment. Additionally, we provided normative data in percentile ranks. In sum, our findings provide three significant contributions to stress research: (1) confirmation that BASS effectively captures distinct dimensions of stress beliefs with robust psychometric properties in a representative sample; (2) evidence that the bifactor model offers superior fit compared to the established three‐factor structure, suggesting refinements to theoretical conceptualization of stress beliefs; and (3) demonstration of measurement invariance across demographic groups, establishing the scale's broad applicability. Importantly, the presented results confirmed hypothesized association suggesting beliefs in stress controllability and positive stress beliefs corresponded with healthy psychological outcomes (e.g., sense of coherence, internal locus of control), whereas negative stress beliefs about stress aligned with poorer mental health outcomes (e.g., depression, anxiety).

Our findings on convergent measures extend cognitive appraisal theory by demonstration how specific dimensions of stress beliefs relate to established psychological constructs. The revealed significant positive correlation between stress controllability, positive stress beliefs, and favorable mental health outcomes provides empirical support for the theoretical models positing that cognitive appraisals serve as mediators between objective stressors and health outcomes (Gaab 2009; Junker et al. 2021). This findings align with the “stress‐is‐enhancing” mindset, which conceptualizes a constructive view of stress with better performance, enhanced health, and psychological resilience, as well as an internal locus of control (Keech et al. 2018; J. A. Laferton et al. 2023; J. A. C. Laferton et al. 2024; Uzun and Karatas 2020; Yeager et al. 2022). These results support this theoretical framework by showing that individuals who perceive stress as manageable and potentially beneficial are better equipped to handle stressors, reinforcing adaptive responses. Similarly, our findings also showed that negative stress beliefs correlate with poorer mental health outcomes (e.g., depression, anxiety), corroborating previous studies that link negative perceptions of stress to adverse health effects (Nabi et al. 2013; Keller et al. 2012). This suggests that viewing stress as inherently harmful may exacerbate its impact on mental health, potentially heightening vulnerability to stress‐related conditions. Altogether, these findings highlight the importance of fostering a constructive mindset toward stress to support psychological well‐being.

The findings concerning the factorial validity revealed that hat while the three‐factor structure of the BASS demonstrated acceptable fit, the bifactor model exhibited superior psychometric properties—a finding that holds important theoretical implications for conceptualizing stress beliefs. The emergence of a strong general factor alongside specific dimensions suggests that stress beliefs operate simultaneously at two cognitive levels: (1) a global evaluative framework through which individuals generally interpret stress experiences, and (2) specific belief domains that influence particular aspects of stress response. This bifactor structure aligns with and extends hierarchical models of cognitive appraisal (Smith and Lazarus 1993; Moors 2013) by empirically demonstrating that stress beliefs specifically exhibit both general and dimension‐specific properties. Further, the BASS effectively captures nuanced dimensions of stress beliefs, particularly emphasizing negative stress beliefs as the most distinct and reliable factor, explaining 32% of item variance beyond the general factor. This factor provides empirical support for theoretical propositions that negative appraisals may constitute especially potent cognitive mechanisms in stress processes (Nabi et al. 2013; Keller et al. 2012). This methodological refinement enables more accurate investigation of how distinct stress belief components uniquely contribute to health outcomes. Moreover, the strong reliability of each scale (i.e., *Negative Stress Beliefs, Controllability, and Positive Stress Beliefs*) supports its use in both clinical and research settings supported by the provision of norm values. In addition, AVE and HTMT values were satisfactory, indicating good convergent and discriminant validity between the BASS subfactors. However, the improved fit of the bifactor model indicated that, while the three factors are distinct, they also share a general underlying construct of stress beliefs. This means that practitioners can use the BASS to assess both the overarching belief about stress and the specific dimensions—negative, controllable, and positive beliefs—depending on their research or clinical focus. For research purposes the bifactorial model is recommended because of its improved model fit, offering more precise measurement by separating general evaluative tendencies from specific belief dimensions. In addition to the strong general factor, the substantial variance in *Negative Stress Beliefs* indicates this dimension may be particularly useful for understanding maladaptive stress perceptions in populations with higher stress or mental health challenges. This aligns with the existing literature (e.g. J. A. C. Laferton et al. 2020; J. A. Laferton et al. 2023; J. A. C. Laferton et al. 2024; Fischer et al. 2016), in which negative stress beliefs are more consistently linked to health outcomes than positive stress beliefs or controllability beliefs. The weak reliability, accordingly low factor loadings, and lack of convergent validity of the specific factors should be noted as a potential drawback of the bifactor model. The Controllability and Positive subscales should thus be interpreted with caution, if at all. They may also represent method and wording effects that don't carry strong predictive value regarding the hypothesized underlying construct. For applied settings, this model provides a reliable and multidimensional approach for evaluating stress beliefs, with the flexibility to focus on both global and specific aspects, depending on the needs of the study or intervention.

With reference to measurement invariance the confirmatory factor and bifactor analyses of the BASS reveal key insights into its measurement stability across sex and age groups. Confirmatory analysis showed that threshold invariance holds across both models, suggesting comparable interpretation of scale points. Loading invariance was consistent for sex comparisons but questionable across age groups, especially in the bifactor model, indicating potential age‐related differences in item interpretation. The bifactor model presented more notable deviations, with ΔCFI and χ^2^‐test results highlighting sensitivity to group differences, while the correlated factors model remained more stable. This suggests that the bifactor model's inclusion of both general and specific factors may capture subtle group‐specific variances that the correlated factors model overlooks. Latent mean comparisons across groups indicated small‐to‐moderate effect sizes, supporting meaningful but not substantial group differences. These results suggest that while BASS scores are generally reliable across demographics, minor adjustments may be useful in age‐specific interpretations. In practical terms, the findings validate the BASS's cross‐group applicability while highlighting age‐specific considerations, particularly for bifactor modeling.

A central contribution of this study is the establishment of population‐based normative values for the BASS, addressing a significant gap in stress assessment methodology. These normative values enhances the utility of the BASS by enabling (1) standardized cross‐study comparisons previously hindered by the absence of normative benchmarks, (2) allowing practitioners to contextualize individual scores within broader population trends and (3) effectively tailor interventions, (4) evaluation of treatment effects in stress management programs. Given the significant associations we documented between stress beliefs and mental health outcomes, these normative data provide a foundation for developing stress belief modification interventions that potentially constitute a preventive approach to stress‐related disorders. Our findings particularly suggest that interventions targeting negative stress beliefs may yield the most substantial clinical benefits, given this dimension's strong association with psychological distress and its emergence as the most distinct factor in bifactor analyses.

### 4.1 Strengths and Limitations

A key strength of this study is its large, representative sample, which significantly enhances the generalizability of findings across diverse populations. This robust sample size allows for more reliable conclusions about the instrument's performance across varied demographic groups. Further, the provision of population‐based normative values is a significant contribution to the practical applicability of the BASS in clinical and research contexts. However, while this study offers substantial methodological advances, several limitations warrant consideration. The norm values we provide are based on a three‐factor model, though our data indicate that a bifactor model better represents the construct. This discrepancy reflects the current transition in understanding stress belief dimensionality. Researchers wishing to employ the bifactor model will therefore need to conduct confirmatory factor analyses to establish model fit and ensure accurate interpretation, as bifactor‐specific norms were not developed in this study. Second, the cross‐sectional design precludes causal inferences regarding the relationship between stress beliefs and mental health outcomes. The absence of longitudinal data limits our ability to demonstrate the stability of stress beliefs over time and their predictive validity for health outcomes. Future studies could address these limitations by employing the BASS in longitudinal designs to explore causal relationships between beliefs about stress and stress appraisal and burden. Moreover, while this sample is representative, cross‐cultural validation could be of interest as the current validation was conducted exclusively in a German sample, restricting its transcultural validity. Finally, integration of the BASS with objective health parameters and physiological stress markers would further establish its criterion and biological validity and strengthen connections to psychoneuroendocrinological models of stress.

## 5 Conclusion

This study validated the BASS in a representative German sample, confirming acceptable reliability, validity, and applicability providing measurement invariance for age and sex. Positive stress beliefs and perceived controllability demonstrated significant positive correlations with adaptive psychological functioning. Conversely, negative stress beliefs exhibited robust associations with adverse mental health indicators. These findings underscore the fundamental role of constructive stress appraisals in promoting psychological resilience.

Factorial validity of the three‐factor structure was questionable and should be re‐evaluated by future research. The bifactor model fit significantly better, suggesting an overarching construct of stress beliefs. However, the specific factors were of limited use. Normative data provided are practical for individualized assessments and may also inform the design of indicative interventions by identifying individuals with particularly negative stress beliefs based on cutoff scores. In summary, the present study provides the first psychometrically validated measure of stress beliefs with normative data, establishing the BASS as both a robust research instrument and the first tool suitable for individual diagnostic applications. The availability of population‐based norms significantly enhances the clinical utility of the scale. By establishing the reliability and validity of the BASS in a representative general population sample, this work extends beyond prior studies limited to selected or convenience samples. This advancement lays a solid foundation for broader applications of stress belief assessment in both scientific research and clinical practice.

## Author Contributions

**Bjarne Schmalbach:** writing – original draft, writing – review and editing, methodology, formal analysis. **Ileana Schmalbach:** writing – original draft, writing – review and editing, methodology, formal analysis. **Johannes A. C. Laferton:** writing – original draft, writing – review and editing, conceptualization. **Bernhard Strauß:** writing – review and editing, data curation. **Jörg M. Fegert:** writing – review and editing, data curation. **Elmar Brähler:** writing – review and editing, data curation. **Katja Petrowski:** writing – review and editing, data curation.

## Conflicts of Interest

The authors declare no conflicts of interest.

## Data Availability

The data that support the findings of this study are available from the corresponding author upon reasonable request.
